# Notes for authors

**DOI:** 10.1155/S1463924686000032

**Published:** 1986


prepared laboratory packages, has succeeded in establishing the concept of the computer as an essential tool in the practice of chemistry in the majority of the students. The aversion to numerical techniques commonly displayed by part-time chemistry students has been largely overcome and most now feel comfortable using sophisticated methods of data processing.

The success of the current programme of work in establishing favourable student attitudes to laboratory computing is demonstrated by the suggestions for extension into other areas now being received from the students. The aim of the next phase is to improve the data-handling techniques now in use and to extend them for further applications. The preliminary work in instrumental control must be consolidated, pending the purchase of further electronically operated instruments. Perhaps the greatest challenge to the staff is to develop the available techniques with sufficient rapidity to keep abreast of the increasing experience of computing of students now entering the Department's courses.

### References

1. Lee, J. D. and Lee, T. D., *Statistics and Computer Methods in BASIC* (Von Nostrand Reinhold, 1982).
2. Norris, A. C., *Computational Chemistry* (John Wiley and Sons, 1981).

### ANALYTICA 86

*3 to 6 June 1986 at the Munich Trade Fair Centre*

The scientific programme for this international meeting is divided into symposia, posters and the 'Analytica – Forum München' (in this latter sector, exhibiting companies will present papers on developments in industrial research). Topics to be covered include:

Separation methods
- Chromatographic methods
- Electrophoretic methods

Combined methods: MS-MS, HPLC-MS, GC-MS, GC-IR, LC-MS, LC-NMR, FT-GC, GC-FTIR
Emission spectroscopy
Bioluminescence, chemiluminescence, fluorimetry
NMR, its application *in vivo*
Radiochemical procedures
Topochemical procedures
Enzymatic analysis
Cell and organ culture based analysis
Dry support reagents including stick tests
Progress in development of reference methods.

*Enquiries about the commercial exhibition and registration to Münchener Messe- und Ausstellungsgesellschaft mbH, Analytica 86, Postfach 12 10 09, D 8000 München 12, FR Germany. Information about scientific contributions from Professor Dr H. Feldman, Inst. für Physiologische Chemie der Universität, Goethestrasse 33, D 8000 München 2, FR Germany.*

### NOTES FOR AUTHORS

*Journal of Automatic Chemistry incorporating Journal of Clinical Laboratory Automation* covers all aspects of automation and mechanization in analytical, clinical and industrial environments. The *Journal* publishes original research papers; short communications on innovations, techniques and instrumentation, or current research in progress; reports on recent commercial developments; and meeting reports, book reviews and information on forthcoming events. All research papers are refereed.

### Manuscripts

Two copies of articles should be submitted. All articles should be typed in double spacing with ample margins, on one side of the paper only. The following items should be sent: (1) a title-page including a brief and informative title, avoiding the word 'new' and its synonyms; a full list of authors with their affiliations and full addresses; (2) an abstract of about 250 words; (3) the main text; (4) appendices (if any); (5) references; (6) tables, each table on a separate sheet and accompanied by a caption; (7) illustrations (diagrams, drawings and photographs) numbered in a single sequence from 1 upwards and with the author's name on the back of every illustration; captions to illustrations should be typed on a separate sheet. Papers are accepted for publication on condition that they have been submitted only to this *Journal*.

### References

References should be indicated in the text by numbers following the author's name, i.e. Skeggs [6]. In the reference section they should be arranged thus:

to a journal

Manks, D. P., *Journal of Automatic Chemistry*, **3** (1981), 119.

to a book

Malmstadt, H. V., in *Topics in Automatic Chemistry*, Ed. Stockwell, P. B. and Foreman, J. K. (Horwood, Chichester, 1978), p. 68.

### Illustrations

Original copies of diagrams and drawings should be supplied, and should be drawn to be suitable for reduction to the page or column width of the *Journal*, i.e. to 85 mm or 179 mm, with special attention to lettering size. Photographs may be sent as glossy prints or as negatives.

### Proofs and offprints

The principal or corresponding author will be sent proofs for checking and will receive 50 offprints free of charge. Additional offprints may be ordered on a form which accompanies the proofs.

*Manuscripts should be sent to either Dr P. B. Stockwell or Ms M. R. Stewart, see inside front cover.*

